# 高效液相色谱-四极杆/静电场轨道阱高分辨质谱法快速分析三子散的入血成分及代谢产物

**DOI:** 10.3724/SP.J.1123.2021.09022

**Published:** 2022-07-08

**Authors:** Huiwen ZHANG, Huimin XIA, Hong LIU, Yanyan LIU, Xin JIU, Minhui ZHANG, Chunlong HE, Huanyun WANG

**Affiliations:** 内蒙古医科大学药学院, 内蒙古 呼和浩特 010110; College of Pharmacy, Inner Mongolia Medical University, Hohhot 010110, China

**Keywords:** 高效液相色谱, 四极杆/静电场轨道阱高分辨质谱, 三子散, 血清药物化学, high performance liguid chromatography (HPLC), quadrupole/electrostatic field orbitrap high resolution mass spectroscopy (Q/Orbitrap HRMS), Sanzi San, serum pharmacochemistry

## Abstract

蒙药三子散由诃子、川楝子、栀子3味药材等比例组成,其临床用药主要采用口服给药方式,药物在体内的吸收、分布、代谢、排泄过程与药物发挥药理作用和疗效的产生密切相关,因此考察灌胃给药后的入血成分有助于阐明三子散的药效物质基础。研究采用血清药物化学研究思路,将Wistar大鼠分成空白组和给药组,给药组给予三子散水提物,腹主动脉取血,离心制备血清样品,采用高效液相色谱-四极杆/静电场轨道阱高分辨质谱(HPLC-Q/Orbitrap HRMS),在SHIMADZU GIST C_18_色谱柱(150 mm×4.6 mm, 5 μm)上进行色谱分离;以甲醇和0.1%(v/v)甲酸水溶液为流动相进行梯度洗脱,柱温30 ℃,流速0.5 mL/min,进样量10 μL,采用加热电喷雾电离(HESI)源,正、负离子同时扫描。通过比对三子散含药血清和空白血清的图谱差异,查阅数据库、各类成分体内代谢途径、三子散成分的相关文献,采用Xcalibur 3.0软件进行峰提取、峰匹配等质谱数据处理,结合Compound Discover 3.0软件对化合物代谢途径的预测分析和裂解规律的推断,解析三子散水提液经大鼠灌胃后血清中的原型成分和代谢产物。结果表明,在给药大鼠血清样品中鉴定出55种入血成分,其中41种原型成分,14种代谢产物。入血的原型成分主要为鞣质类、环烯醚萜类和小分子酚酸类。该研究较为全面地阐释了三子散水提液在大鼠血中的移行成分,有助于揭示三子散的药效物质基础,为该药的临床应用提供参考。

## Abstract (en)

Sanzi San, a Mongolian medicine, comprises three herbs: *Terminalia chebula*, *Melia toosendan*, and *Gardenia jasminoides*. Clinically, Sanzi San is administered orally and distributed via blood to the action site, which implies that the absorption, distribution, metabolism, and excretion (ADME) are closely related to the pharmacological action and curative effect. Therefore, possible explanations for the material basis of Sanzi San were explored in this study preliminarily. A strategy based on serum pharmacochemistry was first applied to explore the absorbed bioactive components and metabolites of Sanzi San. Wistar rats were randomly divided into normal and dosing groups, which were provided with the Sanzi San’s water extract for three days. Then, the rat’s blood samples were obtained from their abdomiral aorta using a sterile blood collection tube after administering the medicine. The blood samples were then centrifuged at 3500 r/min for 10 min to obtain the serum samples.

A practical method based on high performance liquid chromatography coupled with quadrupole and electrostatic field orbitrap high resolution mass spectrometry (HPLC-Q/Orbitrap HRMS) was developed to screen and analyze numerous bioactive components and metabolites adsorbed in the serum of the dosing rats after oral administration of the Sanzi San’s water extract. Chromatographic separation was achieved on a SHIMADZU GIST C_18_ chromatographic column (150 mm×4.6 mm, 5 μm). The temperature of the column was maintained at 30 ℃. The flow rate was 0.5 mL/min, and the injection volume was 10 μL. The mobile phase comprised an aqueous solution of 0.1% formic acid and methanol under gradient elution. A heated electrospray ion (HESI) source was used with positive and negative ion scanning modes. To rapidly screen out and identify the absorbed bioactive components and metabolites of Sanzi San in the rat serum samples, a simple three-step approach was developed. First, the known components in Sanzi San were listed systematically by exploring various databases, such as the Web of Science, PubMed, and Chinese National Knowledge Infrastructure. In addition, relevant information on drug biotransformation and the characteristic fragmentation patterns of parent compounds were summarized. Second, the absorbed components and metabolites were ascertained using the Xcalibur 3.0 software. Based on the information related to the parent compound’s structure, the software could be used to identify the unique peaks by comparing the chromatograms of the normal and dosing samples. Consequently, the total ion chromatograms of serum samples were established. Finally, the Compound Discover 3.0 software was used to predict the metabolic pathways and fragmentation of the absorbed compounds. Using this approach, 55 compounds were characterized, including 41 prototype components and 14 metabolites. The main prototype components in the serum sample were tannins, iridoids, and phenolic acids. The details of these compounds have been summarized and presented. Regarding the absorbed bioactive components and metabolites in the serum samples of rats administered with Sanzi San, phase Ⅰ and phase Ⅱ biochemical reactions were involved in the biotransformation pathways. The phase Ⅰ reaction modified the components and created sites for the phase Ⅱ reaction, involving reduction and hydrolysis. The phase Ⅱ reaction coupled groups to existing conjugation sites, including glucuronide to glucuronic acid, sulfate, and methyl. MS/MS spectra indicated that methylation, demethylation, and dehydroxylation are the metabolic pathways of procyanidins. Additionally, glucuronidation, deglucosidation, hydration, and demethylation are the metabolic pathways of iridoids in Sanzi San. This study comprehensively analyzed the components of the Sanzi San’s water extract absorbed in the rat’s serum. Our results revealed information regarding the pharmacodynamic substances and the major pathways involved in the ADME of Sanzi San. Further, potential medicinal ingredients for the pharmacological effects and clinical use of Sanzi San were explored at the serum pharmacochemistry level.

蒙药复方制剂三子散,蒙文名为图喜木勒-3,由诃子、川楝子、栀子3味药材等比例组成,最早收载于《四部医典》,现收载于2020版《中国药典》中^[1]^,具有清热凉血、解毒的功效,蒙医用于治疗与血、热、湿有关的疾病,临床上常用于治疗高血压、高血脂和动脉粥样硬化等^[2]^,蒙医中还采用三子散结合放血疗法用于高脂血症的治疗。三子散临床用药主要采用口服给药方式,而药物在体内的吸收、分布、代谢、排泄过程,与药物发挥药理作用和疗效的产生密切相关。因此,考察灌胃给药后的入血成分有助于阐明三子散的药效物质基础。而目前三子散的研究仅关注于其主要化学成分含量的研究,缺少对三子散的体内代谢和药效物质基础研究。

中药是一个多种类多成分综合作用的复杂体系,其中的有效成分需要吸收入血,并作用于相应靶点才能发挥整体药效作用。因此王喜军发展并提出了“中药血清药物化学”研究思路^[3,4]^,即采用高效液相色谱-质谱联用技术(HPLC-MS),以给药后的动物血清为研究对象,通过比对药材中的成分、空白血清以及含药血清进行鉴定分析,明确血中移行成分,结合化合物的质谱信息快速、精准地筛选出药效成分,为进一步阐明复方制剂的药效物质基础奠定基础。HPLC-MS同时兼备了高效液相色谱的分离手段和质谱检测功能,具有高灵敏、高分辨、高通量的优势^[5]^,这使得它在药物代谢转化的研究过程中,对药物成分和代谢产物的鉴定分析发挥了重要作用。

本课题组前期建立了三子散HPLC指纹图谱及一测多评分析方法^[6⇓-8]^,并通过文献检索及前期实验,发现三子散中的化合物包括鞣质、酚酸、环烯醚萜、单萜、三萜、有机酸酯类成分等,但尚未明确入血成分及其体内代谢途径。因此,本实验拟采用高效液相色谱-四极杆/静电场轨道阱高分辨质谱(HPLC-Q/Orbitrap HRMS),对照已鉴定的三子散复方中的化学成分,分析大鼠体内三子散入血原型、代谢成分及化学成分的质谱裂解规律,建立一种快速有效分析三子散入血成分及其代谢途径的分析方法,为进一步阐明三子散的药效物质基础和体内动态变化规律提供研究依据。

## 1 实验部分

### 1.1 仪器、试剂与材料

Q-Exactive高效液相色谱-四极杆静电场轨道阱高分辨质谱仪、UltiMate 3000超高效液相色谱仪(美国Thermo公司); AB 135-S型十万分之一分析天平(瑞士Metter Toledo公司); KQ-250 DA型数控超声波清洗器(昆山市超声仪器有限公司); WH-1微型涡旋混合仪(上海沪西分析仪器厂); 3K15低温高速离心机(德国Sigma公司)。

没食子酸(批号:110831-200302,中国药品生物制品检定所);鞣花酸(批号:C10024692,上海麦克林生化科技有限公司);没食子酸甲酯(批号:A1810031)、莽草酸(批号:G1509047)、没食子酸乙酯(批号:E119029)和奎宁酸(批号:Q109705)均购自上海阿拉丁试剂有限公司;甲醇(色谱纯,美国Thermo公司);实验用水为超纯水。

诃子为使君子科植物诃子(*Terminalia chebula* Retz.)的干燥成熟果实(批号:18042801,产地:广西);栀子为茜草科栀子(*Gardenia jasminoides* Ellis.)的干燥成熟果实(批号:171001429,产地:江西);川楝子为楝科植物川楝子(*Melia toosendan* Sieb. et Zucc.)的干燥成熟果实(批号:170801,产地:四川);性状均符合2020年版《中国药典》(一部)的规定。

Wistar大鼠,体重范围200~250 g,购买于内蒙古医科大学动物中心,于内蒙古医科大学实验动物中心普通环境饲养。

### 1.2 对照品溶液的制备

分别取对照品没食子酸、鞣花酸、没食子酸甲酯、莽草酸、没食子酸乙酯、奎宁酸适量,精密称定,加甲醇制成200 mg/L的对照品溶液,12000 r/min离心5 min后取上清,即得。

### 1.3 给药样品的制备

取诃子、栀子、川楝子三味药材粉碎,过40目筛,等量混匀,称取200 g三子散粉末,蒸馏水超声提取3次,每次1 h,合并提取液,减压浓缩后,冷冻干燥制成三子散冻干粉。

称取50 g三子散粉末,加入50 mL 0.5%羧甲基纤维素钠,配制成质量浓度为1 g/mL的三子散溶液。

### 1.4 血清样品的采集

取12只Wistar大鼠,随机分成空白组和给药组,适应性饲养1 w,实验前禁食12 h,全程不禁水。给药组给予5 mL三子散溶液,空白组给予5 mL蒸馏水,连续灌胃3 d,最后一次给药1 h后,腹主动脉取血,3500 r/min离心10 min后取上清,-80 ℃冰箱储存备用,空白血清收集方式相同。

### 1.5 血清样品的处理

分别吸取1 mL空白组血清和三子散给药血清,加入3 mL甲醇沉淀蛋白质,涡旋1 min后,12000 r/min离心取上清,氮吹,加入200 μL甲醇,涡旋1 min复溶后,12000 r/min离心后取上清,HPLC-Q/Orbitrap HRMS检测。

### 1.6 色谱和质谱条件

色谱柱:SHIMADZU GIST C_18_色谱柱(150 mm×4.6 mm, 5 μm);柱温:30 ℃;进样量:10 μL;流速:0.5 mL/min;流动相A: 0.1%(v/v)甲酸水溶液,流动相B:甲醇。梯度洗脱程序:0~10 min, 1%B~15%B; 10~30 min, 15%B~30%B; 30~45 min, 30%B~35%B; 45~75 min, 35%B~47%B; 75~85 min, 47%B~85%B; 85~95 min, 85%B~90%B。

离子源:加热电喷雾电离(HESI)源;正离子和负离子模式同时扫描;喷雾电压:4000 V(ESI^+^)/3500 V(ESI^-^);碰撞能量(CE):45 eV(ESI^+^)和30 eV(ESI^-^);辅助气体体积流量:30 L/min;离子传输管温度:300 ℃;辅助气温度:100 ℃;检测方式为全扫描/数据依赖二级扫描(Full-MS/dd-MS^2^)模式;Full MS分辨率70000, dd-MS^2^分辨率17500;扫描范围*m/z* 100~1500。

### 1.7 三子散体内入血成分及代谢产物的鉴定

查阅数据库、各类成分体内代谢途径、三子散成分的相关文献,采用Xcalibur 3.0软件进行峰提取、峰匹配等质谱数据处理,结合Compound Discover 3.0软件对化合物代谢途径的预测分析和裂解规律的推断,收集整理三子散入血成分及代谢产物。

## 2 结果与讨论

### 2.1 实验条件优化

#### 2.1.1 色谱-质谱条件的优化

三子散中化学成分复杂,结构特征繁杂,差异较大,其中,诃子中的酚酸类成分在负离子模式下易发生去质子化^[9]^,而单萜和环烯醚萜类成分在正离子模式下易发生质子化^[10]^,故兼顾不同成分特性选用正、负离子两种模式进行检测。实验对液相色谱条件进行了优化,当流动相中不加甲酸时,以没食子酸为代表的酚酸类成分出现峰分离度低及峰形拖尾等现象,加入甲酸后,这种情况消失,因此选用0.1%(v/v)甲酸水溶液-甲醇作为流动相。

#### 2.1.2 样品前处理条件的优化

三子散在临床上是通过口服给药,因此本实验模拟人体的给药方式,选择水作为提取溶剂,并比较回流和超声两种提取方式,结果发现超声提取更完全,由于提取溶剂为水,加热回流的温度要求较高,可能将药材中部分受热不稳定的成分破坏,故选择超声提取方式及冷冻干燥方式浓缩。

血清样品处理中,分别采用甲醇和乙腈进行血清蛋白质的沉淀,乙腈沉淀后的血样不能完全和血样进行混合,无法将蛋白质完全沉淀出来,影响化学成分的鉴定,而甲醇可与血清均匀混合并沉淀完全,因此选择甲醇沉淀蛋白质。

### 2.2 三子散原型成分鉴定

运用HPLC-Q/Orbitrap HRMS技术对空白组及给药组大鼠血清进行分析,采用Compound Discover软件对其化学成分进行快速识别及分析鉴定,根据保留时间、精确质量数、碎片离子峰和前期三子散化学成分鉴定结果进行分析鉴定。给药后血清和空白血清的图谱见图1。三子散给药血清中鉴别出55种入血成分,其中31种来自诃子^[11,12]^, 23种来自栀子^[13,14]^, 1种来自川楝子^[15]^。其中含有41种原型成分,包括9种鞣质、15种酚酸类、7种环烯醚萜、1种三萜、4种有机酚酸和5种单萜类成分,鉴定结果见表1。

**图 1:**
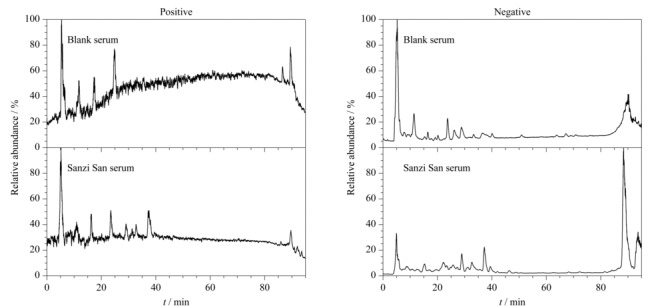
正、负离子模式下空白血清和三子散给药血清的总离子流图

**表 1:** 大鼠血清中的原型成分

No.	Compound	*t*_R_/ min	Ion mode	Predicated ion (*m/z*)	Observed ion (*m/z*)	Product ions (*m/z*)	Error					Source	
1^*^	quinic acid	5.07	[M-H]^-^	191.05501	191.05504	173.04425,127.03856,111.04348,	0.025					H	
						93.03301							
2	3,4-dihydroxy benzaldehyde	5.83	[M-H]^-^	137.02332	137.02315	109.02799	-0.171					Z	
3^*^	shikimic acid	5.90	[M-H]^-^	173.04444	173.04411	155.03439,137.02261,111.04358	-0.340					H	
4	chebulic acid	8.80	[M-H]^-^	355.02958	355.03018	337.01953,293.03137,249.03989,	0.592					H	
						205.04962,161.05936							
5	6-*O*-galloyl-glucose	12.44	[M-H]^-^	331.06597	331.06674	271.04562,169.01276,125.02287	0.767					H	
6	2-*O*-galloyl-glucose	13.22	[M-H]^-^	331.06597	331.06653	271.04510,169.01279,125.02314	0.557					H	
7	1-*O*-galloyl-glucose	14.51	[M-H]^-^	331.06597	331.06625	271.04547,169.01285,125.02318	0.277					H	
8^*^	gallic acid	15.09	[M-H]^-^	169.01314	169.01274	125.02285	-0.410					H	
9	5-hydroxymethylfurfural	15.21	[M-H]^-^	125.02332	125.02302	78.91732	-0.301					C	
10	chebulic acid/isomer	16.32	[M-H]^-^	355.02958	355.03030	337.11356,293.01355,248.97058,	0.622					H	
						204.98022,161.04433							
11	13-methylchebulate/11-methylchebulate	17.65	[M-H]^-^	369.04523	369.04581	353.03043,337.01788,205.04951	0.572					H	
12	3,6-di-*O*-galloyl-*β*-D-glucose	19.44	[M-H]^-^	483.07693	483.07724	331.06665,313.05609,211.06020,	0.308					H	
						169.08574							
13	shanzhiside	20.92	[M-H]^-^	391.12634	391.12399	229.07060,185.08072,167.06992	0.502					Z	
14	(-)-shikimide 4-*O*-gallate/(-)-shikimide	21.13	[M-H]^-^	325.05540	325.05612	169.01286,125.02290, 93.03308	0.712					H	
	3-*O*-gallate/(-)-shikimide 5-*O*-gallate												
15	protocatechuic acid	22.01	[M-H]^-^	153.01823	153.01823	109.02800	-0.365					Z	
16	(-)-shikimide 4-*O*-gallate/(-)-shikimide	22.40	[M-H]^-^	325.05540	325.05631	169.01286,125.02287, 93.03308	0.712					H	
	3-*O*-gallate/(-)-shikimide 5-*O*-gallate												
17	1,4-di-*O*-galloyl-*β*-D-glucose	23.48	[M-H]^-^	483.07693	483.07782	331.06778,313.05704,211.02417,	0.888					H	
						169.01332							
18	(-)-shikimide 4-*O*-gallate/(-)-shikimide	23.66	[M-H]^-^	325.05540	325.05569	169.01286,125.02291, 93.03300	0.282					H	
	3-*O*-gallate/(-)-shikimide 5-*O*-gallate												
19	shanzhiside methyl ester	24.44	[M-H]^-^	405.13913	405.13913	359.13603,197.08096	0.722					Z	
20	uralenneoside	25.51	[M-H]^-^	285.06049	285.06131	153.01840,109.02811	0.816					H	
21	1,3-di-*O*-galloyl-*β*-D-glucose	25.85	[M-H]^-^	483.07693	483.07828	331.96085,313.05597,210.99361,	1.348					H	
						169.08578							
22	jasminoside B/D/G	26.63	[M+H]^+^	347.17004	347.16989	167.10651		-0.154		Z		
23^*^	methyl gallate	29.42	[M-H]^-^	183.02879	183.02875	168.00449,124.01493,111.00724		-0.201		H		
24	jasminodiol/rehmapicrogenin	30.48	[M-H]^-^	183.10157	183.10149	139.11147		-0.081		Z		
25	1,6-di-O-galloyl-β-D-glucose	30.63	[M-H]^-^	483.07693	483.07797	331.06635,313.05630,211.02336,		1.038		H		
						169.01770						
26	chlorogenic acid	32.50	[M-H]^-^	353.08670	353.08741	191.05504		0.691		Z		
27	genipin-1,10-di-O-β-D-glucopyranoside	32.68	[M+H]^+^	551.19704	551.19696	227.09093,209.08066,149.05962		-0.087		Z		
28	jasminoside S/I	34.75	[M+H]^+^	493.22795	493.22809	207.06659,169.12222		0.137		Z		
29	jasminoside S/I	36.25	[M+H]^+^	493.22795	493.22754	169.12224		-0.143		Z		
30	genipin	37.13	[M+H]^+^	227.09140	227.09093	209.08058		-0.470		Z		
31	geniposide	37.48	[M+H]^+^	389.14422	389.13971	227.09077,209.08055,125.09614		-4.513		Z		
32^*^	ethyl gallate	42.64	[M-H]^-^	197.04444	197.04605	169.01393,125.02393,110.94225		1.600		H		
33	trans-phenylacrylic acid	46.37	[M-H]^-^	163.03917	163.03871	119.04740		-0.261		H		
34	jasminodiol/rehmapicrogenin	46.53	[M-H]^-^	183.10157	183.10153	139.11150		-0.041		Z		
35	10-O-trans-sinapoylgeniposide/	56.33	[M-H]^-^	593.18648	593.18842	225.14905, 207.13829		1.938		Z		
	6'-O-trans-sinapoylgeniposide											
36	3,4-dihydroxy benzaldehyde/isomer	58.69	[M-H]^-^	137.02332	137.02304	109.02830		-0.281		Z		
37^*^	ellagic acid	68.95	[M-H]^-^	300.99789	300.99857	283.75159		0.676		H		
38	4-O-sinapoyl-5-O-caffeoylquninic acid	71.13	[M-H]^-^	559.14461	559.14624	397.91110, 173.04422		1.623		Z		
39	4-O-sinapoyl-5-O-caffeoylquninic acid	78.73	[M-H]^-^	559.14461	559.14626	397.11221, 173.04428		1.743		Z		
	/isomer											
40	10-O-trans-sinapoylgeniposide/6'-	79.32	[M-H]^-^	593.18648	593.18842	225.14919,207.13840		1.938		Z		
	O-trans-sinapoylgeniposide											
41	arjungenin	90.61	[M-H]^-^	503.33671	503.33945	485.32883		2.734		H		

* Confirmed by standard substance; H: *Terminalia chebula* Retz.; Z: *Gardenia jasminoides* Ellis.; C: *Melia toosendan* Sieb. et Zucc.

### 2.3 三子散入血相关代谢产物及代谢途径

三子散入血的原型成分主要有鞣质类、环烯醚萜类和小分子酚酸成分,这几类成分在体内主要代谢途径包括I相代谢(还原反应、水解反应)和Ⅱ相代谢(甲基化、磺酸酯化和葡萄糖醛酸化),血清中的代谢物主要来自于诃子和栀子,共有14种,其中9种来源于诃子,5种来源于栀子,结果见表2,提取离子流图和质谱图谱见图2。

**表 2:** 大鼠血清中的三子散代谢产物

No.	Compound	t_R_/ min	Ion mode	Predicated ion (m/z)	Observed ion (m/z)	Product ions (m/z)	Elemental composition	Error	Source
M1	quinic acid reduction	5.05	[M-H]^-^	193.07066	193.07069	191.05502,129.01772	C_7_H_14_O_6_	0.025	H
M2	urolithin D	12.10	[M-H]^-^	259.02371	259.01004	191.05370,179.05345	C_13_H_8_O_6_	3.674	H
M3	digallic acid hydration	15.31	[M-H]^-^	339.03467	339.03522	169.01280,125.02287	C_14_H_12_O_10_	0.547	H
M4	gallic acid glucuronidation	18.83	[M-H]^-^	345.04523	345.04590	169.01286,125.02292	C_13_H_14_O_11_	0.662	H
M5	shanzhiside methyl ester reduction	20.07	[M-H]^-^	407.15478	407.15546	361.14996,199.09650	C_17_H_28_O_11_	0.672	Z
M6	3,4-dihydroxy benzaldehyde sulfation	21.02	[M-H]^-^	216.98013	216.98027	137.02298	C_7_H_6_O_6_S	0.195	H
M7	10-O-acetylgeniposide reduction	21.82	[M-H]^-^	419.11840	419.11917	397.94940,211.06027,	C_17_H_24_O_12_	0.768	Z
						167.06990			
M8	jasminodiol/rehmapicrogenin	26.01	[M-H]^-^	359.13365	359.13367	183.02872	C_16_H_24_O_9_	0.011	H
	glucuronidation								
M9	methyl gallate reduction	27.40	[M-H]^-^	183.02879	183.02859	168.00502,124.01509	C_8_H_8_0_5_	-0.210	H
M10	shikimic acid reduction	28.76	[M-H]^-^	175.06009	175.05978	157.04913,113.05927	C_7_H_12_O_5_	-0.320	H
M11	genipin glucuronidation	37.49	[M+H]^+^	403.12348	403.12344	209.08057	C_17_H_22_O_11_	1.052	Z
M12	genipin demethylation	70.33	[M+H]^+^	212.06792	212.06816	210.15672,127.07531	C_10_H_11_O_5_	0.235	Z
M13	6'-O-trans-sinapoyl jasminoside	81.15	[M+H]^+^	539.24868	539.24890	363.21616,327.19510	C_27_H_38_O_11_	-0.028	Z
	A reduction								
M14	dimethyl ellagic acid	86.62	[M-H]^-^	329.02919	329.03027	314.00638,298.98288	C_16_H_10_O_8_	1.076	H

**图 2:**
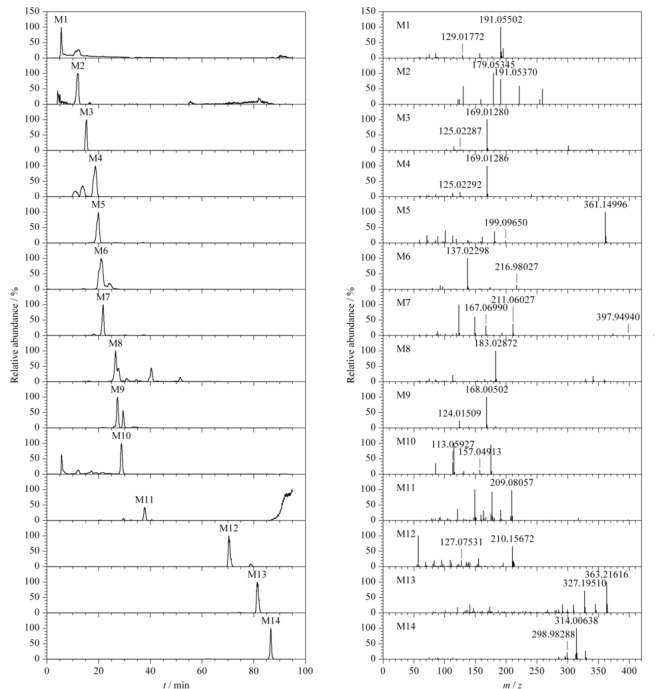
三子散代谢产物的提取离子流色谱图和质谱图

#### 2.3.1 鞣质类化合物的代谢途径

在大鼠血清中检测鞣质类有3种代谢途径:甲基化、脱甲基+脱羟基和脱羟基。以鞣花酸(ellagic acid)为例简析鞣质类化合物裂解规律,见图3a。在负离子模式下进行一级全扫描,与空白血清比较,灌胃给药后的大鼠血清样品中检测到鞣花酸的分子离子峰(*m/z* 300.99857),代谢物M14(*t*_R_=86.62 min)产生*m/z* 329.03027 [M-H]^-^分子离子峰,相对分子质量比鞣花酸多28 Da,提示其为鞣花酸的甲基化代谢产物,初步推测为二甲基鞣花酸(dimethyl ellagic acid);代谢物M2(*t*_R_=12.10 min)产生*m/z* 259.01004 [M-H]^-^分子离子峰,相对分子质量比鞣花酸少42 Da,推测其可能发生了脱甲基和脱羟基代谢过程,推测该化合物为尿石素(urolithin D)。

**图 3:**
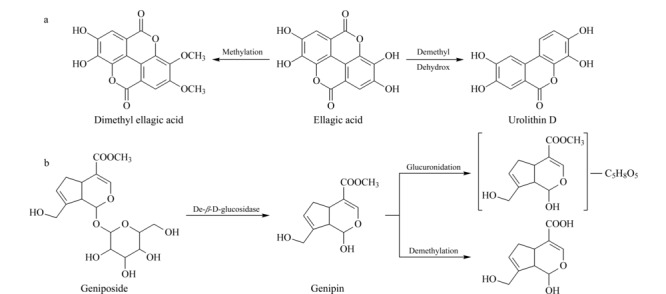
(a)鞣花酸和(b)京尼平在大鼠体内的代谢途径

#### 2.3.2 环烯醚萜类化合物的代谢途径

在大鼠血清中检测环烯醚萜类有4种代谢途径:葡萄糖醛酸化、脱*β*-D-葡萄糖苷、水合化、脱甲基。以京尼平(genipin)为例简析环烯醚萜类化合物的裂解规律,见图3b。在正离子模式下进行一级全扫描,检测到京尼平的分子离子峰(*m/z* 227.09093);京尼平苷(geniposide)通过*β*-D-葡萄糖苷酶分解成京尼平;代谢物M11(*t*_R_=37.49 min)产生*m/z* 403.12344 [M+H]^+^分子离子峰,相对分子质量比京尼平多了176 Da,提示该化合物是通过葡萄醛酸化代谢过程产生的京尼平葡萄糖醛酸化产物;代谢物M12(*t*_R_=70.33 min)产生*m/z* 212.06816 [M+H]^+^分子离子峰,相对分子质量比京尼平少15 Da,推测其为京尼平的脱甲基化产物(genipin demethylation)。

## 3 结论

本实验通过建立HPLC-Q/Orbitrap HRMS快速分析了三子散的入血成分及代谢产物,并结合相关文献进行入血成分分析对比,在大鼠给药血清中,指认出55种入血成分,其中41种原型成分,14种代谢产物。这些成分是复方中与药理作用相关的化学成分,有助于阐明三子散的药效物质基础,并为其临床合理应用提供参考依据。
